# Trace Metals in Cannabis Seized by Law Enforcement in Ghana and Multivariate Analysis to Distinguish among Different Cannabis Farms

**DOI:** 10.3390/toxics10100567

**Published:** 2022-09-27

**Authors:** Chris Douvris, Edward Bentil, Isaac Ayensu, Clement Osei Akoto, Isaac Kingsley Amponsah, Joseph Adu, Derek Bussan

**Affiliations:** 1 Theobald Science Center, Department of Biological and Chemical Sciences, New York Institute of Technology, Old Westbury, NY 11568, USA; 2 Department of Pharmaceutical Chemistry, Faculty of Pharmacy and Pharmaceutical Sciences, College of Health Sciences, Kwame Nkrumah University of Science and Technology, Kumasi, Ghana; 3 Department of Chemistry, College of Sciences, Kwame Nkrumah University of Science and Technology, Kumasi, Ghana; 4 Department of Pharmacognosy, Faculty of Pharmacy and Pharmaceutical Sciences, College of Health Sciences, Kwame Nkrumah University of Science and Technology, Kumasi, Ghana; 5 Department of Chemistry, Eastern Kentucky University, 521 Lancaster Ave, Richmond, KY 40475, USA

**Keywords:** toxic metals, ICP-MS, cannabis, soil, forensic science, illicit drugs

## Abstract

For hundreds of years, cannabis has been one of the most known cultivated plants due to its variety of uses, which include as a psychoactive drug, as well as for medicinal activity. Although prohibiting cannabis products, the countries of the African continent are the largest producers of cannabis in the world; a fact that makes the trafficking of cannabis-based illicit drugs a high priority for local law enforcement authorities. The latter are exceedingly interested in the use of chemical analyses for facilitating quantification, identification, and tracing of the origin of seized cannabis samples. Targeting these goals, and focusing on the country of Ghana, the present study used inductively coupled plasma mass spectrometry (ICP-MS) for the determination of 12 elements (Pb, Cu, Ca, Mg, Mn, Zn, Cd, As, Hg, Fe, Na, and K) in cannabis seized by Ghana’s law enforcement authorities and soils of cannabis farms. Furthermore, multivariate analysis was applied to distinguish among different cannabis farms and match them with the samples. As a result, 22 seized cannabis samples and 12 other cannabis samples with their respective soils were analyzed to reveal considerable As and Pb concentrations. As and Pb levels in cannabis were found up to 242 ppb for As and 854 ppb for Pb. Multivariate analysis was applied for separating different cannabis farms and seized samples based on elemental analysis, evidently linking the seized samples with two Ghana regions.

## 1. Introduction

Cannabis, which is also widely known by the name marijuana, is an herbal psychoactive drug that derives from the Cannabis plant [1]. The plants contain chemicals include cannabinoids that attach to specific sites in the brain and on the nerves, meaning the drug has been commonly used both recreationally and medicinally for centuries [2]. Although the use of cannabis is prohibited in most countries, including Ghana, it has recently undergone explosive growth as several countries, including several states in the United States, have passed legislation that approves both its recreational and medical use [3]. Ghana, along with Nigeria, is one of the top illicit cannabis-producing countries of West Africa, and as a result, it is a matter of high importance to study the presence of toxic metals and metalloids of Ghana cannabis samples. The United Nations Office on Drugs and Crime (UNODC) estimates that almost 4% of the global population aged 15–64 years have used cannabis at least one time in 2019, which translates to 200 million people, while these numbers are expected to increase dramatically and reach 11% of the population or more than half a billion people by 2030 [4]. A direct outcome of these staggering numbers for cannabis users is the expansion of the plant’s producers, which, according to UNODC, have increased by fourfold from 1995 to 2019 [4].

The psychoactive effects of cannabis on the brain are widely known, and they stem from the organic constituent compounds (such as delta-9-tetrahydrocannabinol and cannabidiol), which are thoroughly investigated. Nevertheless, there is an extensive variation on the elemental content, including toxic metal and metalloid content, among different cannabis plants, depending on where the plants are grown [5]. It is primarily the toxicity of certain metals and metalloids, such as arsenic and lead, that make elemental investigations of cannabis varieties an area of paramount importance, but also a number of other important considerations such as the characterization or association of a particular cannabis sample with its origin area, and the help of law-enforcing agencies of countries that prohibit the plant to trace the source of the drug [6,7]. Cannabis-containing toxic metals and metalloids that are consumed in combustive form can be of a great health danger as this toxicity in the human body takes place via the production of reactive oxygen species and free radicals, which can damage enzymes, proteins, lipids and nucleic acids, and cause cancer and neurological issues [8].

A number of different analytical techniques, as well as the interpretation of their data such as chemometrics, have been used for probing the geographical sources of plants and soils [9,10,11]. Among them, inductively coupled plasma mass spectrometry (ICP-MS) has been the method of choice for the determination of toxic metals and metalloids of plants and soils, with low detection limits down to 0.1 ppb, simple sample preparation, high throughput and the ability to measure many elements simultaneously [10,12,13,14,15,16,17,18,19].

Considering the high importance of assessing the elemental content of cannabis and associating it with a particular geographical region, and focusing on cannabis originating from the country of Ghana, we set out to determine 12 elements (Pb, Cu, Ca, Mg, Mn, Zn, Cd, As, Hg, Fe, Na, and K) in seized cannabis-producing Ghana regions earmarked by the Narcotic Control Commission (NACOC) and the drug law enforcement unit of the Ghana Police Service. A total of 34 seized marijuana samples along with 12 soil samples from three farms were digested and analyzed via ICP-MS to reveal considerable As and Pb concentrations. Moreover, as multivariate analysis is a powerful technique to separate out samples and geographical areas by chemometrics [20,21], multivariate analysis was applied for separating different cannabis farms and seized samples based on elemental analysis, clearly associating the seized samples with two particular Ghana regions.

## 2. Materials and Methods

Materials. Samples for the study were taken from illegal farms located in two regions of Ghana, namely the Eastern and Bono Regions. Two farms were found in two different towns, Nsawkaw and Badu in the Bono region, and one in the Eastern area, located specifically at Boti. A total of 12 cannabis samples were taken from the two regions, 6 from the Eastern part and 6 from the Bono region, with their corresponding soil samples. The sample plan followed is summarized in Tables S1 and S2 (Supplementary). All sampled plants were grown from seeds and collected at three months of harvesting. Fertilizers were applied as declared by the farmers. Samples collected were immediately placed in a rubber pack and taken to an open place. They were dried for two months at room temperature. The flowers and leaves were separated from the seeds and processed for analysis. 22 seized cannabis samples from 2017 to 2020 were also analyzed. Ultra-pure grade chemicals were used, and ultra-pure water was prepared by the Ghana Standards Authority, Accra, Ghana. Argon gas was purchased from Ghana Gas Company, Accra, Ghana and used as received for ICP-MS. 

Methods. The cannabis and soil samples were put in rubber holders, properly sealed, and accordingly labeled. Each sample was a cluster consisting of 4 different locations in a quadrant. All cannabis samples were accompanied with corresponding soil samples. The cannabis and soil samples were kept at a temperature of 30 °C and 75% humidity with all security protocols in place. An analytical balance with capacity of 220 g from the Ghana Standards Authority Forensic Lab was used to weigh the dry cannabis and soil samples. Calibration of the electronic balance was done using standard weights. All 34 samples were homogenized with Fritsch planetary ball mill. 

For the digestion of the cannabis samples: 250 mg of the sample was weighed in a mineralization Teflon vessel, 3 mL of 65% nitric acid (Suprapur; Merck, Darmstad, Germany) and 1 mL of 30% hydrogen peroxide (Suprapur) was added. Next, samples were placed in a high-pressure MULTIWAVE sample preparation system made by Anton Paar (Perkin Elmer, Waltham, MA, USA). Table S3 presents the sample digestion program. Next, samples were quantitatively transferred into 10 mL (class A, Brand) flasks, and diluted up to the mark with deionized water (Milli Q, Billerica, MA, USA). 

The method by Falciani et al. was followed for the analysis of soil samples [22]. Accordingly, 0.1 g of each of the 12 soil samples were weighed into a closed vessel for digestion. 5 mL of HNO_3_ were added into the digestion vessels, followed by 3 mL of HF, and 1 mL of H_2_O. The vessels were then sealed for digestion in a microwave operating at 250 W for 10 min. After digestion, the solution was cooled down to room temperature and then transferred into a 100 mL volumetric flask. It was finally diluted to mark using deionized H_2_O. 

Preparation of standard solutions. Using a pipette, 1 mL was drawn from the standard stock solution and diluted with 2% HNO_3_ to prepare standard solutions; Standard A, Standard B, and Standard C, were made with concentrations of 200 ppb, 400 ppb, and 600 ppb, respectively. A calibration check was constructed using the 3 standard solutions. For the metals used, stock standard solutions of 99.99% purity of 1000 mg/L of Pb, Cu, Ca, Mg, Mn, Zn, Cd, As, Hg, Fe, Na, and K were also used. All the soil samples and blank solutions were prepared following the same procedure as for the cannabis samples,

ICP-MS Instrumentation. An Agilent 7700 with standard nebulization ICP-MS instrument was used. A total of 12 metals were determined Lead, Copper, Calcium, Magnesium, Manganese, Zinc, Cadmium, Arsenic, Mercury, Iron, Sodium and Potassium, following the ICP-MS parameters that are listed in Table S4 (Supplementary). 

Analytical results were calculated using linear calibration graphs. The concentrations of the metals and metalloids were determined in ppm (µg/g) or ppb (ng/g) using the formula below as reported previously by Bentil et al. [19].

(1) $$\mathrm{Content}=\frac{\left(C-B\right)V}{W}$$

C—Concentration of final solution, B—Concentration of blank solution
V—Final volume of solution, W—Weight of sample

## 3. Results and Discussion

**Figure 1:**
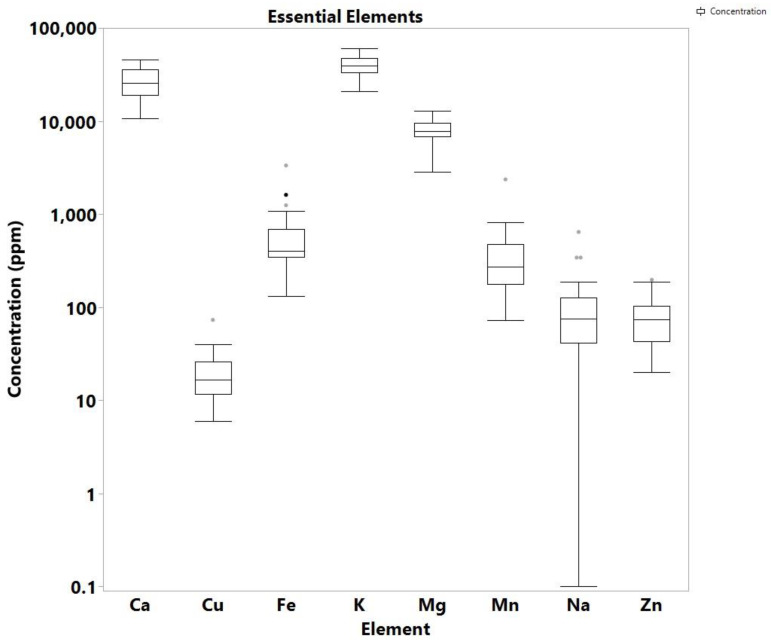
Box plot of essential elements concentration in cannabis samples.

**Figure 2:**
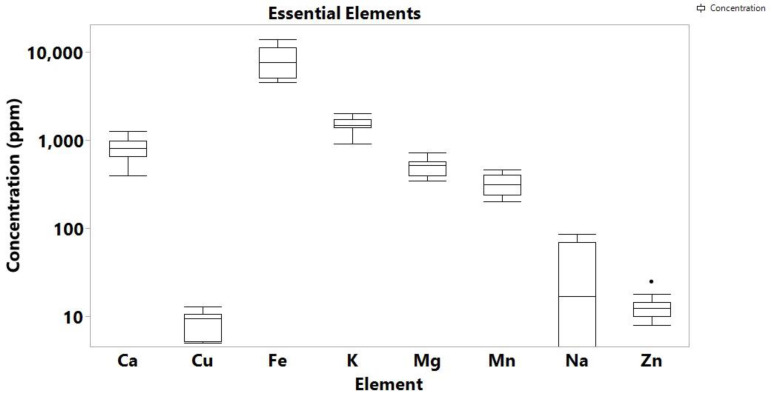
Box plot of essential elements concentration in soil samples.

**Figure 3:**
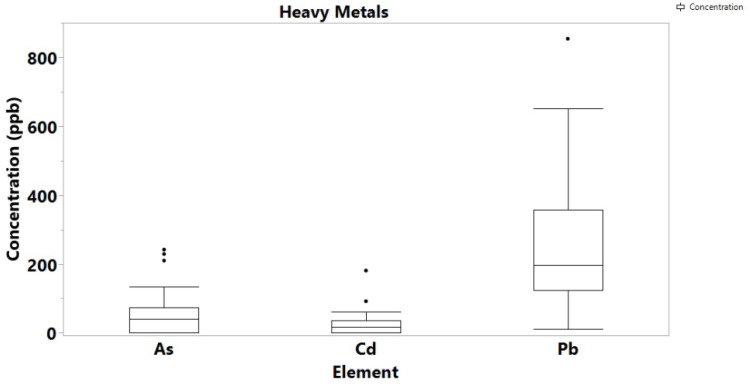
Box plot of toxic metals/metalloids concentration in cannabis samples.

**Figure 4:**
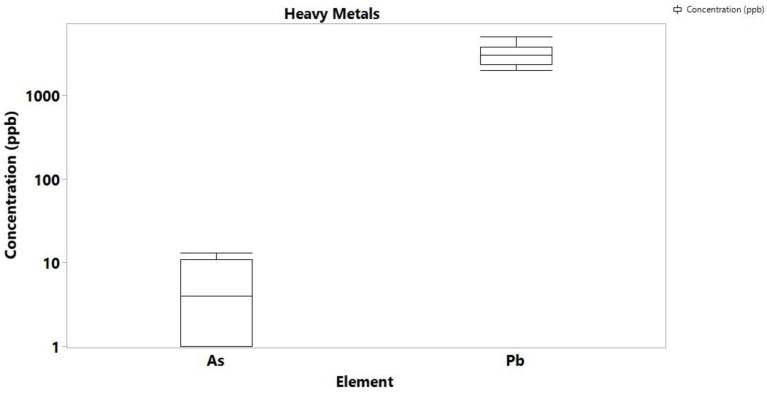
Box plot of toxic metals/metalloids concentration in soil samples.

The quantitative analysis of 12 elements (Pb, Cu, Ca, Mg, Mn, Zn, Cd, As, Hg, Fe, Na, and K) in a total of 46 samples, of which 34 samples were Cannabis samples and 12 soil samples associated with the cannabis regions, was carried out using ICP-MS. The soil and cannabis farm samples all came from Nsawkaw, Badu, and Boti areas in Ghana. While the high concentration of eight of the analyzed elements (Cu, Ca, Mg, Mn, Zn, Fe, Na, K) is not concerning as they are essential for both the plant and the soil, four of the analyzed elements (Hg, Pb, As, Cd) are highly toxic metals and metalloids, which are all known to be carcinogenic, as well as to produce a number of serious adverse health effects. The distribution of the detected elements and the skewness are shown in Figure 1, Figure 2, Figure 3 and Figure 4, while the individual concentrations for each element for both cannabis and soil samples is illustrated in Tables S5–S7 (Supplementary). The levels of concentrations for the eight essential elements in cannabis ranged from non-detectable (N.D.) to 643 ppm for Na, 2830 to 12,939 ppm for Mg, 20,772 to 60,537 ppm for K, 10.699 to 45,652 Ca, 73 to 2363 ppm for Mn, 131 to 3335 ppm for Fe, 6 to 73 ppm for Cu and 20 to 197 ppm for Zn.

These values are comparable to values for cannabis leaves published in other studies. For example, a study by Zafeiraki et al. reported Cannabis samples from various regions in Greece with analogous concentrations: 7.1 to 19.8 thousand ppm for Mg, 24.0 to 123.2 thousand ppm for Ca, 76.8 to 518 ppm for Mn, 135 to 1338 ppm for Fe, 8.2 to 64.2 ppm for Cu and 23 to 157 ppm for Zn [23]. As in the study of Greece, the predominant elements from those analyzed in Ghana cannabis samples was Ca and K, followed by Mg, Mn and Fe. Of interest are the high levels recorded for Fe, which is because most of the cannabis farms are in high rocky areas with rocks containing high levels of the element. During the breakdown of the rocks, Fe is known to enter the soil. The farmers often choose these areas because they want to hide from security agencies. Ca was recorded as the highest metal/metalloid concentration in the cannabis samples for Nsawkaw, Badu, and Boti, with Boti recording the highest, which is consistent with data reported by Kuras and Wachowicz [24]. In addition, all the three farms showed the presence of Cu, Fe, Mg, Mn and Zn, as reported by Shibuya et al. [25], proving that Cannabis sativa can absorb metals and metalloids from the soil irrespective of where it was cultivated, but the levels may be dependent on natural and anthropogenic sources [26].

Moreover, the soils from the Cannabis cultivation regions were analyzed to associate their levels of trace metals/metalloids to cannabis. As a result, the concentrations of the essential elements under study in the 12 soil analyzed samples ranged as follows: up to 86 ppm for Na, 351 to 729 ppm for Mg, 921 to 2042 ppm for K, 400 to 1277 ppm for Ca, 204 to 467 ppm for Mn, 4536 to 14,007 ppm for Fe, 5 to 13 ppm for Cu and 8 to 25 ppm for Zn (Table S4).

With the exemption of Hg of which no detectable concentrations were found in both cannabis samples and soils, the concentration levels of the highly toxic Cd, As and Pb were determined at high levels, but again, they were comparable to previous studies in Cannabis samples from different geographical regions. In particular, Pb ranged from 11 to 854 ppb, As up to 242 ppb, and Cd was determined up to 181 ppb (Figure 3). For comparison, Zafeiraki et al. reported concentrations of 0.01 to 0.85 for Pb, 0.01 to 0.1 ppm for As and 0.003 to 0.18 ppm for Cd. The concentration of Pb in the soil samples ranged from 2.0 to 5.0 ppm, As ranged from 1.2 to 13 ppb, while Cd and Hg were not detectable in any soil samples (Figure 4). Other literature reports on Pb, Cd and As concentrations on cannabis report values averaging 0.5 ppm indicating that the values determined in this study are high but comparable to the literature. 

Of the amounts recorded for the highly toxic metals, Pb concentration was the highest and can harm anyone who takes Cannabis contaminated to this level. Pb contamination in the environment arises from both naturally occurring reasons as it is a component in the earth’s crust, and human activities such as mining, burning of fossil fuels and manufacturing. Particularly for soil, it is known that Pb particles from anthropogenic activities settle on soil and can last for years [27]. In Ghana, Pb is ubiquitous in soil, especially near mining areas, industrial sites, incinerators, farms, landfills, and waste sites. The farms from which samples were collected are in close proximity to landfills, which explains the high levels of the toxic metal [28]. The toxic metal is carcinogenic, and Pb poisoning very effectively occurs by inhalation. In concentrations higher than 0.1 ppm, Pb is known to affect the gastrointestinal tract and the central nervous system, causing a variety of adverse effects on the human organs, particularly to kidneys [29]. 

The presence of Cd in cannabis originates from atmospheric Cd emissions, metal production, sewage mud, fertilizers, and disposal of batteries and other Cd-containing sources [30]. CdO also exists as small particles in the air (fumes) resulting from smelting, soldering, or other high-temperature industrial processes. Since the soil from Boti had no Cd content, but the plants had, it can be suggested that the metal source is airborne. Table S6 shows the level of Cd recorded for 22 seized cannabis samples from 2017 to 2020. Out of the 22 samples, only 2 showed no amount of Cadmium. The three towns used for this research are known for illegal cultivation and big bust of Cannabis by the security agencies. With cadmium level as a varying factor between the visited farms in Bono and Eastern regions, the seized Cannabis can be grouped according to the presence or absence of Cadmium in them. This can be a preliminary way of determining the source of Cannabis when seized. The toxic metal is a known carcinogen which affects the skeletal, urinary, reproductive, cardiovascular, central and peripheral nervous, and respiratory systems. Although, the presence of Hg in Ghana soils has been recorded in the literature, neither the soil areas under study, nor the cannabis samples contained any detectable concentrations.

The amounts of As in both cannabis samples and soils also raises concern as the toxicity of arsenic depending on exposure dose, frequency, duration, biological species, age, and gender, as well as on genetic and nutritional factors, can be very dangerous with cancer known as one of the long-term environmental exposure to it [31,32].

**Figure 5:**
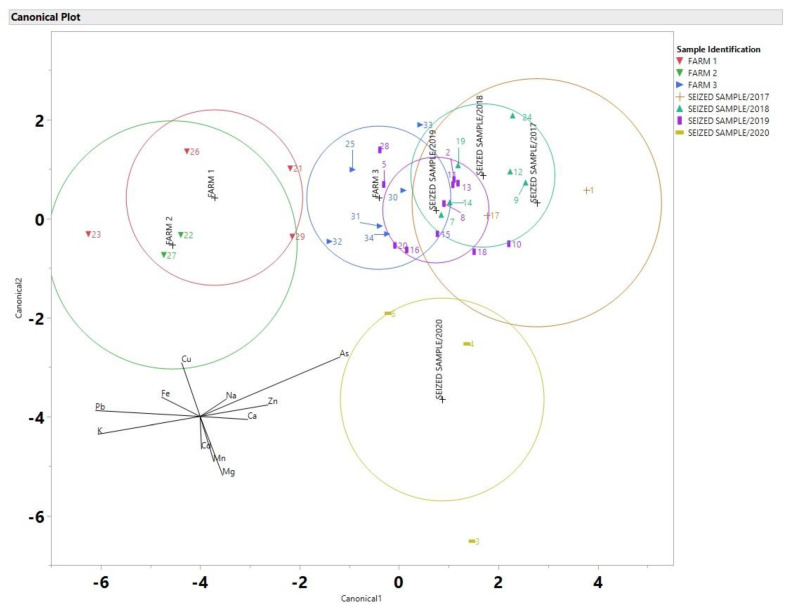
Linear Discriminant (DA) canonical plot using data for 11 elements determined by ICP-MS of the cannabis samples (sample labeling can be found in Table S1).

As Discriminant analysis (DA) has been used by others as a possible technique to link a suspect to a crime scene [11,21,33], it was applied to the data from this study in order to observe if DA is appropriate for a real-world scenarios and in particular for case of seized by the police cannabis samples. For the discriminant analysis plots all data were processed the same, using JMP 14. Data was normalized by using z-scores. As a result, DA is depicted for the cannabis samples using 11 elements in Figure 5. The data set includes data from Farms 1, 2 and 3 as well as data from all the seized samples. Due to the nature of the samples (police seized) the locations are kept confidential. 

As it is suggested by the DA analysis, the seized samples from the year 2020 do not overlap with any of the three farms, this indicates that there is a high probability that these samples did not come from any of the three farm areas. Since Farms 1 and 2 did not have any overlap with any of the seized samples they can be excluded from the possibility that they came from these sites. Farm 3 had the most overlap with the seized samples, and overlapped with the years 2017, 2018 and 2020. Six samples from 2019 overlapped with the ellipsoid of farm 3. A comparison of percent differences is shown in Table 1.

(2) $$\mathrm{Percent}\;\mathrm{difference}\;\frac{\left|a-b\right|}{\frac{a+b}{2}}*100$$

**Figure 6:**
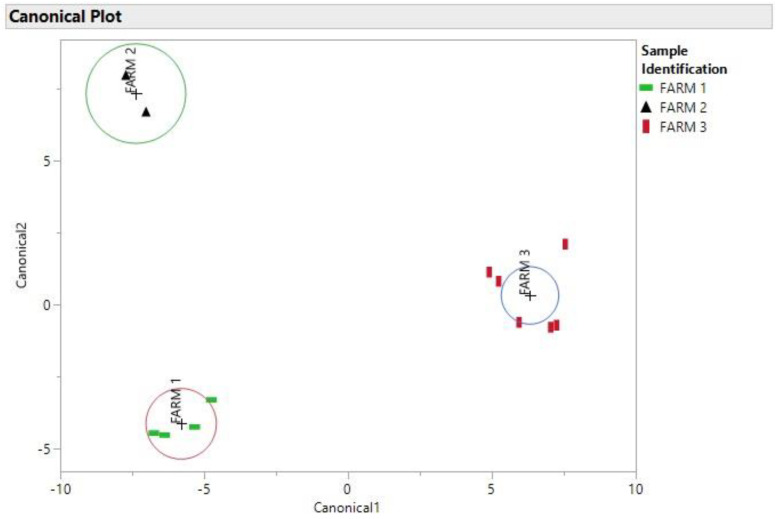
Linear Discriminant (DA) canonical plot using data for 11 elements determined by ICP-MS of the Farm soil samples.

As it is evident, the elements that had the smallest percent difference in Table 1 between samples 34 and 20 were Mg, K, Cu, Zn, As and Cd. Samples 34 and 20 had nearly identical amounts of Mg, As and Cd. Even though samples 8 and 14 were taken in two different years, there is a high probability that these samples could have come from the same area based on their location in the DA plot in Figure 4 and the percent differences in Table 1. Even samples that come from the same farm (samples 31 and 34) have varying percent differences in elemental composition as demonstrated in Table 1, as it can be seen in Figure 6 the DA of the soil samples that shows the soil samples were better separated and this could be due to the fact that the soil samples have a more consistent elemental concentration compared to the cannabis samples which included flowers and leaves.

## 4. Conclusions

The present study investigates the concentrations of trace metals and metalloids in both cannabis samples seized by law enforcement and soils of cannabis farms in Ghana. ICP-MS analysis reveals high concentrations of Pb and As in the cannabis samples, and at the same time, it is illustrated that illicit cannabis is confirmed to contain metals that cannabis plants usually absorb from the soil they are cultivated on. Moreover, the ICP-MS metal profile on the seized cannabis samples can identify cannabis that comes from the same city or region even though they might be from different seizures. 

In particular, multivariate analysis was used to distinguish among different cannabis farms and match them with the samples. The DA plot was able to separate out the three farms under study based on the elemental analysis, as well as different farms and seized samples based on elemental analysis. Future studies should include a higher number of samples and different geographical locations.

As one of the drawbacks of using multivariate analysis methods is sample size, future studies could also increase sample size to make more accurate predications regarding authentication.

Finally, as the seized samples did not contain any soil samples, future seized samples would benefit from taking soil samples as the soil samples had better separation on the DA plot. 

## Figures and Tables

**Table 1:** Percent difference between selected sample sites.

	Na	Mg	K	Ca	Mn	Fe	Cu	Zn	As	Cd	Pb
percent Δ (samples 34 and 20)	104.8	0.1	16.0	54.6	77.0	54.6	20.0	10.5	1.7	0.0	37.5
percent Δ (samples 8 and 14)	42.1	3.6	0.2	40.4	33.4	128.5	28.6	4.8	131.3	147.2	124.4
percent Δ (samples 31 and 34)	161.0	0.3	0.6	5.9	20.7	2.8	25.0	42.7	58.0	28.6	27.6

## Data Availability

Data is available upon request.

## Supplementary Materials

The following supporting information can be downloaded at: https://www.mdpi.com/article/10.3390/toxics10100567/s1, Table S1. Sample plan employed for the cannabis and soil samples in Bono Region. Table S2. Sample plan employed for the cannabis and soil samples in Eastern Region. Table S3. Microwave digestion operational parameters. Table S4. ICP-MS operational parameters. Table S5. Essential Elements Cannabis Sample Concentrations in (µg g^−1^). Table S6. Heavy Metals Cannabis Sample Concentrations in (ng g^−1^). Table S7. Soil Sample Concentrations in (µg g^−1^), As and Pb (ng g^−1^).
