# Supplementary material for: Trace Metals in Cannabis Seized by Law Enforcement in Ghana and Multivariate Analysis to Distinguish among Different Cannabis Farms
Source: Toxics. 2022 Sep 27;10(10):567. doi: 10.3390/toxics10100567 (PMC9612285; doi:10.3390/toxics10100567)
Supplement: Supplementary file 1 [file toxics-10-00567-s001.zip › toxics 191558-suppl.pdf]

**Table S1.** Sample plan employed for the cannabis and soil samples in Bono Region.

| Nsawkaw | | Badu | |
|---|---|---|---|
| **Cannabis Samples Code** | **Soil Samples Code** | **Cannabis Samples Code** | **Soil Samples Code** |
| BAN(C1) | BAN(C1s) | BAN(Cb) | BAN(Cbs) |
| BAN(C2) | BAN(C2s) | BAN(Cc) | BAN(Ccs) |
| BAN(C3) | BAN(C3s) | BAN(Cd) | BAN(Cds) |

BAN - Bono region; C – cluster; s - soil.

**Table S2.** Sample plan employed for the cannabis and soil samples in Eastern Region.

| Boti | |
|---|---|
| **Cannabis samples code** | **Soil samples code** |
| EAS(C1) | EAS(C1s) |
| EAS(C2) | EAS(C2s) |
| EAS(C3) | EAS(C3s) |
| EAS(C4) | EAS(C4s) |
| EAS(C5) | EAS(C5s) |
| EAS(C6) | EAS(C6s) |

BAN - Eastern region; C – cluster; s - soil.

**Table S3.** Microwave digestion operational parameters.

| **Step** | Power (W) | Time (min) |
|---|---|---|
| 1 | 100-300 | 0-5 |
| 2 | 0 | 6-7 |
| 3 | 300-600 | 8-13 |
| 4 | 0 | 14-19 |
| 5 | 600-800 | 20-21 |
| 6 | 0 | 22-23 |
| 7 | 800-1000 | 24-29 |
| 8 | 0 | 30-31 |
| 9 | 1000 | 32-37 |

**Table S4.** ICP-MS operational parameters.

| **Parameters** | **Conditions** |
|---|---|
| RF Power | 1 kW |
| Radio frequency | 40 MHz |
| Carrier gas flow rate | 0.64L/min |
| Plasma gas flow rate | 15 L/min |
| Plasma observation | Radial |
| Pump uptake | 1.5 ml/min |

**Table S5.** Essential Elements Cannabis Sample Concentrations in ($\mu g\ g^{-1}$).

| Sample Type | Sample Name | SAMPLE Code Name | Sample Number | Na | Mg | K | Ca | Mn | Fe | Cu | Zn |
|---|---|---|---|---|---|---|---|---|---|---|---|
| Cannabis | SEIZED SAMPLE/2017 | 10/FS2/17 | Sample 1 | 335 | 9717 | 60537 | 33631 | 763 | 851 | 20 | 100 |
| Cannabis | SEIZED SAMPLE/2019 | 39/FS2/19 | Sample 2 | 127 | 12660 | 39777 | 45652 | 460 | 3335 | 40 | 186 |
| Cannabis | SEIZED SAMPLE/2020 | 10/FS2/20 | Sample 3 | 84 | 12939 | 47664 | 31696 | 2363 | 453 | 28 | 85 |
| Cannabis | SEIZED SAMPLE/2020 | 11/FS2/20 | Sample 4 | 127 | 11596 | 47351 | 40098 | 768 | 392 | 26 | 96 |
| Cannabis | SEIZED SAMPLE/2019 | 22/FS2/19 | Sample 5 | N.D. | 4303 | 34626 | 16493 | 284 | 270 | 9 | 37 |
| Cannabis | SEIZED SAMPLE/2020 | 9/FS2/20 | Sample 6 | 110 | 9961 | 51607 | 36916 | 494 | 378 | 16 | 70 |
| Cannabis | SEIZED SAMPLE/2018 | 15/FS2/18 | Sample 7 | 348 | 9567 | 39447 | 42080 | 645 | 732 | 24 | 139 |
| Cannabis | SEIZED SAMPLE/2019 | 12/FS2/19 | Sample 8 | 45 | 9715 | 34722 | 21095 | 339 | 351 | 40 | 107 |
| Cannabis | SEIZED SAMPLE/2018 | 1/FS2/18 | Sample 9 | 184 | 7537 | 35725 | 38995 | 551 | 1071 | 16 | 71 |
| Cannabis | SEIZED SAMPLE/2019 | 9/FS2/19 | Sample 10 | 91 | 8780 | 34964 | 40887 | 331 | 362 | 24 | 76 |
| Cannabis | SEIZED SAMPLE/2019 | 23/FS2/19 | Sample 11 | 61 | 8279 | 48298 | 36648 | 819 | 1245 | 35 | 118 |
| Cannabis | SEIZED SAMPLE/2018 | 37/FS2/18 | Sample 12 | 110 | 12211 | 47243 | 24860 | 653 | 544 | 73 | 197 |
| Cannabis | SEIZED SAMPLE/2019 | 27/FS2/19 | Sample 13 | 81 | 8577 | 33950 | 25695 | 350 | 711 | 31 | 98 |
| Cannabis | SEIZED SAMPLE/2018 | 16/FS2/18 | Sample 14 | 69 | 9374 | 34784 | 31773 | 475 | 1612 | 30 | 102 |

| | | | | | | | | | | | |
|---|---|---|---|---|---|---|---|---|---|---|---|
| Cannabis | SEIZED SAMPLE/2019 | 44/FS2/19 | Sample 15 | 126 | 8991 | 36245 | 16976 | 258 | 301 | 23 | 95 |
| Cannabis | SEIZED SAMPLE/2019 | 17/FS2/19 | Sample 16 | 109 | 9787 | 48410 | 39851 | 202 | 480 | 16 | 61 |
| Cannabis | SEIZED SAMPLE/2017 | 42/FS2/17 | Sample 17 | 20 | 8066 | 27579 | 25568 | 422 | 368 | 26 | 77 |
| Cannabis | SEIZED SAMPLE/2019 | 13/FS2/19 | Sample 18 | 186 | 8439 | 47166 | 13682 | 272 | 173 | 16 | 160 |
| Cannabis | SEIZED SAMPLE/2018 | 20/FS2/18 | Sample 19 | 80 | 6899 | 31863 | 20363 | 220 | 408 | 26 | 105 |
| Cannabis | SEIZED SAMPLE/2019 | 46/FS2/19 | Sample 20 | 55 | 7811 | 38835 | 20315 | 457 | 681 | 11 | 60 |
| Cannabis | FARM 1 | C3(BAN1) | Sample 21 | 18 | 4885 | 31983 | 19717 | 117 | 464 | 17 | 69 |
| Cannabis | FARM 2 | Cc(BAN2) | Sample 22 | 66 | 7524 | 54220 | 20760 | 151 | 295 | 22 | 62 |
| Cannabis | FARM 1 | C2(BAN1) | Sample 23 | 67 | 6724 | 54897 | 21801 | 124 | 548 | 15 | 43 |
| Cannabis | SEIZED SAMPLE/2018 | 38/FS2/18 | Sample 24 | 643 | 5743 | 26030 | 13862 | 183 | 357 | 11 | 161 |
| Cannabis | FARM 3 | C6(EAS1) | Sample 25 | 31 | 3469 | 27287 | 14700 | 108 | 131 | 10 | 38 |
| Cannabis | FARM 1 | CB(BAN1) | Sample 26 | N.D. | 2830 | 21893 | 10804 | 73 | 421 | 6 | 20 |
| Cannabis | FARM 2 | CD(BAN2) | Sample 27 | 56 | 7495 | 52831 | 28598 | 164 | 495 | 15 | 43 |
| Cannabis | SEIZED SAMPLE/2019 | 14/FS2/19 | Sample 28 | 101 | 5095 | 25500 | 10699 | 270 | 324 | 24 | 83 |
| Cannabis | FARM 1 | C1(BAN1) | Sample 29 | 44 | 7008 | 36358 | 21698 | 178 | 371 | 14 | 40 |
| Cannabis | FARM 3 | C5(EAS1) | Sample 30 | 42 | 4958 | 20772 | 15696 | 233 | 240 | 13 | 44 |
| Cannabis | FARM 3 | C4(EAS1) | Sample 31 | 19 | 7839 | 45355 | 33533 | 165 | 400 | 7 | 35 |
| Cannabis | FARM 3 | C1(EAS1) | Sample 32 | 90 | 7634 | 54588 | 35216 | 175 | 305 | 12 | 43 |
| Cannabis | FARM 3 | C3(EAS1) | Sample 33 | 41 | 7361 | 43748 | 28662 | 188 | 832 | 10 | 37 |
| Cannabis | FARM 3 | C2(EAS1) | Sample 34 | 176 | 7817 | 45611 | 35586 | 203 | 389 | 9 | 54 |

**Table S6.** Heavy Metals Cannabis Sample Concentrations in (ng $g^{-1}$).

| Sample Type | Sample Name | SAMPLE Code Name | Sample Number | As | Cd | Pb | Hg |
|---|---|---|---|---|---|---|---|
| Cannabis | SEIZED SAMPLE/2017 | 10/FS2/17 | Sample 1 | 210 | 34 | 235 | N.D. |
| Cannabis | SEIZED SAMPLE/2019 | 39/FS2/19 | Sample 2 | 79 | 61 | 473 | N.D. |
| Cannabis | SEIZED SAMPLE/2020 | 10/FS2/20 | Sample 3 | 29 | 61 | 86 | N.D. |
| Cannabis | SEIZED SAMPLE/2020 | 11/FS2/20 | Sample 4 | 34 | 41 | 115 | N.D. |
| Cannabis | SEIZED SAMPLE/2019 | 22/FS2/19 | Sample 5 | 75 | 26 | 146 | N.D. |
| Cannabis | SEIZED SAMPLE/2020 | 9/FS2/20 | Sample 6 | 40 | 20 | 124 | N.D. |
| Cannabis | SEIZED SAMPLE/2018 | 15/FS2/18 | Sample 7 | 64 | 29 | 651 | N.D. |
| Cannabis | SEIZED SAMPLE/2019 | 12/FS2/19 | Sample 8 | 23 | 14 | 100 | N.D. |
| Cannabis | SEIZED SAMPLE/2018 | 1/FS2/18 | Sample 9 | 134 | 48 | 357 | N.D. |
| Cannabis | SEIZED SAMPLE/2019 | 9/FS2/19 | Sample 10 | 48 | 44 | 123 | N.D. |
| Cannabis | SEIZED SAMPLE/2019 | 23/FS2/19 | Sample 11 | 242 | 181 | 854 | N.D. |
| Cannabis | SEIZED SAMPLE/2018 | 37/FS2/18 | Sample 12 | 53 | 52 | 159 | N.D. |
| Cannabis | SEIZED SAMPLE/2019 | 27/FS2/19 | Sample 13 | 65 | 33 | 234 | N.D. |
| Cannabis | SEIZED SAMPLE/2018 | 16/FS2/18 | Sample 14 | 111 | 92 | 429 | N.D. |
| Cannabis | SEIZED SAMPLE/2019 | 44/FS2/19 | Sample 15 | 8 | 3 | 11 | N.D. |
| Cannabis | SEIZED SAMPLE/2019 | 17/FS2/19 | Sample 16 | 69 | 9 | 204 | N.D. |
| Cannabis | SEIZED SAMPLE/2017 | 42/FS2/17 | Sample 17 | 42 | 20 | 124 | N.D. |
| Cannabis | SEIZED SAMPLE/2019 | 13/FS2/19 | Sample 18 | 20 | 34 | 16 | N.D. |
| Cannabis | SEIZED SAMPLE/2018 | 20/FS2/18 | Sample 19 | 37 | 17 | 144 | N.D. |
| Cannabis | SEIZED SAMPLE/2019 | 46/FS2/19 | Sample 20 | 61 | 12 | 158 | N.D. |
| Cannabis | FARM 1 | C3(BAN1) | Sample 21 | N.D. | N.D. | 260 | N.D. |
| Cannabis | FARM 2 | Cc(BAN2) | Sample 22 | N.D. | N.D. | 210 | N.D. |
| Cannabis | FARM 1 | C2(BAN1) | Sample 23 | N.D. | N.D. | 367 | N.D. |
| Cannabis | SEIZED SAMPLE/2018 | 38/FS2/18 | Sample 24 | N.D. | N.D. | 361 | N.D. |
| Cannabis | FARM 3 | C6(EAS1) | Sample 25 | N.D. | N.D. | 45 | N.D. |
| Cannabis | FARM 1 | CB(BAN1) | Sample 26 | N.D. | N.D. | 463 | N.D. |
| Cannabis | FARM 2 | CD(BAN2) | Sample 27 | N.D. | N.D. | 273 | N.D. |
| Cannabis | SEIZED SAMPLE/2019 | 14/FS2/19 | Sample 28 | N.D. | N.D. | 152 | N.D. |
| Cannabis | FARM 1 | C1(BAN1) | Sample 29 | N.D. | N.D. | 158 | N.D. |
| Cannabis | FARM 3 | C5(EAS1) | Sample 30 | N.D. | N.D. | 86 | N.D. |
| Cannabis | FARM 3 | C4(EAS1) | Sample 31 | 109 | 16 | 305 | N.D. |
| Cannabis | FARM 3 | C1(EAS1) | Sample 32 | 73 | 22 | 188 | N.D. |
| Cannabis | FARM 3 | C3(EAS1) | Sample 33 | 229 | 35 | 635 | N.D. |
| Cannabis | FARM 3 | C2(EAS1) | Sample 34 | 60 | 12 | 231 | N.D. |

**Table S7.** Soil Sample Concentrations in ($\mu$g g$^{-1}$), As and Pb (ng g$^{-1}$).

| Sample Type | Sample Name | Sample Code Name | Sample Number | Na | Mg | K | Ca | Mn | Fe | Cu | Zn | As | Cd | Pb | Hg |
|---|---|---|---|---|---|---|---|---|---|---|---|---|---|---|---|
| soil | C1(BAN1) | FARM 1 | Sample 1 | 75 | 582 | 2042 | 807 | 444 | 9572 | 11 | 13 | 7 | N.D. | 2646 | N.D. |
| soil | C5(EAS1) | FARM 3 | Sample 2 | 6 | 605 | 1614 | 610 | 297 | 7467 | 5 | 10 | 1 | N.D. | 5017 | N.D. |
| soil | CB(BAN1) | FARM 1 | Sample 3 | 25 | 351 | 921 | 672 | 298 | 14007 | 10 | 9 | 11 | N.D. | 2041 | N.D. |
| soil | C4(EAS1) | FARM 3 | Sample 4 | 9 | 729 | 1756 | 1277 | 356 | 6036 | 10 | 25 | 1 | N.D. | 3490 | N.D. |
| soil | C1(EAS1) | FARM 3 | Sample 5 | 0 | 548 | 1529 | 833 | 361 | 5060 | 8 | 18 | 1 | N.D. | 3086 | N.D. |
| soil | C6(EAS1) | FARM 3 | Sample 6 | N.D. | 503 | 1418 | 809 | 204 | 4984 | 5 | 12 | 1 | N.D. | 4389 | N.D. |
| soil | Cc(BAN2) | FARM 2 | Sample 7 | 36 | 398 | 1539 | 992 | 311 | 7764 | 9 | 10 | 7 | N.D. | 2005 | N.D. |
| soil | C2(BAN1) | FARM 1 | Sample 8 | 86 | 412 | 2033 | 649 | 315 | 12896 | 11 | 10 | 13 | N.D. | 3895 | N.D. |
| soil | C2(EAS1) | FARM 3 | Sample 9 | N.D. | 514 | 1390 | 876 | 221 | 5293 | 6 | 13 | 1 | N.D. | 2977 | N.D. |
| soil | C3(EAS1) | FARM 3 | Sample 10 | N.D. | 393 | 1337 | 400 | 211 | 4536 | 5 | 8 | 1 | N.D. | 3352 | N.D. |
| soil | C3(BAN1) | FARM 1 | Sample 11 | 80 | 545 | 1433 | 1254 | 421 | 10556 | 10 | 13 | 11 | N.D. | 2351 | N.D. |
| soil | CD(BAN2) | FARM 2 | Sample 12 | 54 | 522 | 1464 | 965 | 467 | 11712 | 13 | 15 | 11 | N.D. | 2351 | N.D. |
